# Neuronal Differentiation from Induced Pluripotent Stem Cell-Derived Neurospheres by the Application of Oxidized Alginate-Gelatin-Laminin Hydrogels

**DOI:** 10.3390/biomedicines9030261

**Published:** 2021-03-05

**Authors:** Thomas Distler, Ines Lauria, Rainer Detsch, Clemens M. Sauter, Farina Bendt, Julia Kapr, Stephan Rütten, Aldo R. Boccaccini, Ellen Fritsche

**Affiliations:** 1 Department of Materials Science and Engineering, Institute Biomaterials, University of Erlangen-Nuremberg, Cauerstr. 6, 91058 Erlangen, Germany; rainer.detsch@fau.de; 2 IUF-Leibniz Research Institute for Environmental Medicine, Auf’m Hennekamp 50, 40225 Duesseldorf, Germany; ines.lauria@rwth-aachen.de (I.L.); clemens.sauter@uni-tuebingen.de (C.M.S.); farina.bendt@iuf-duesseldorf.de (F.B.); julia.kapr@iuf-duesseldorf.de (J.K.); 3 Electron Microscopy Facility, Institute of Pathology, RWTH Aachen University Hospital, Pauwelsstrasse 30, 52074 Aachen, Germany; sruetten@ukaachen.de; 4 Medical Faculty, Heinrich-Heine-University, 40225 Düsseldorf, Germany

**Keywords:** oxidized alginate, laminin, hydrogels, human induced pluripotent stem cells (hiPSC), neurospheres, tissue engineering, bioprinting

## Abstract

Biodegradable hydrogels that promote stem cell differentiation into neurons in three dimensions (3D) are highly desired in biomedical research to study drug neurotoxicity or to yield cell-containing biomaterials for neuronal tissue repair. Here, we demonstrate that oxidized alginate-gelatin-laminin (ADA-GEL-LAM) hydrogels facilitate neuronal differentiation and growth of embedded human induced pluripotent stem cell (hiPSC) derived neurospheres. ADA-GEL and ADA-GEL-LAM hydrogels exhibiting a stiffness close to ~5 kPa at initial cell culture conditions of 37 °C were prepared. Laminin supplemented ADA-GEL promoted an increase in neuronal differentiation in comparison to pristine ADA-GEL, with enhanced neuron migration from the neurospheres to the bulk 3D hydrogel matrix. The presence of laminin in ADA-GEL led to a more than two-fold increase in the number of neurospheres with migrated neurons. Our findings suggest that laminin addition to oxidized alginate—gelatin hydrogel matrices plays a crucial role to tailor oxidized alginate-gelatin hydrogels suitable for 3D neuronal cell culture applications.

## 1. Introduction

The fate and organization of stem cells within the human body is dependent on a spatiotemporal exposure to growth factors, cell-cell contacts and physiological cues from the surrounding extracellular matrix (ECM) [1]. Together, these factors constitute the microenvironment or niche of a cell. Traditional in vitro cell cultures are grown in two dimensions and adherent to a flat surface, reflecting the in vivo microenvironment in a simplified manner. Unnatural cell morphology and a general lack of cell–cell interactions, as well as cell-ECM adhesions, represent the limitations of such two-dimensional (2D) models [2]. Depending on the scientific question, 2D cell models can be sufficient to provide a convenient and quick solution for high-throughput applications. However, given the low success rate of drugs targeting brain-related diseases, a need for more complex and physiological neural culture models exists [3,4,5]. Different strategies have emerged to create 3D neural cell models that display higher degrees of structural complexity. Conventional cell biology-based models include spheroids, neurospheres, and organoids, each having their own merits and limitations [6,7,8,9].

While conventional 3D in vitro models might capture the structural and cytoarchitectural aspects of the human brain, they are also prone to variability [10,11]. A key issue regarding the development of consistent cell models is the precise control over their cell and matrix composition as well as spatial organization [12]. Taken together, reproducibility and controllability are critical factors for the implementation of 3D cell models in high-throughput test systems for compound assessment. The mechanical properties of the cells’ environment play a significant role in regulating their behavior [13,14]. The brain is one of the softest tissues in the human body with a Young’s modulus (E) ranging from 0.5 to 50 kPa [15]. In vitro studies have demonstrated that rodent neural stem cells embedded in methacrylated hyaluronic acid (HA) based hydrogels show neuronal differentiation and survival is favored in soft hydrogels (approximately <1 kPa), while astrocytes prefer slightly stiffer gels (<10 kPa) [16,17], which was shown also on 2D peptide-functionalized polyacrylamide (pAAm) hydrogel substrates [16]. In the same context, primary neuronal cells grown on soft (G′ < 300 Pa) substrates exhibited increased neurite branching compared to those grown on stiffer (G′ > 400 Pa) gels [18]. Oxidized alginate, or alginate di-aldehyde (ADA) based ADA-gelatin (ADA-GEL) hydrogel is a promising biomaterial for tissue engineering (TE) as it offers a wide range of tunable properties such as controlled degradation and hydrogel stiffness [19,20]. It is further possible to tune the stiffness and mechanical properties of alginate-gelatin based hydrogels to mimic the stiffness and characteristics of neuronal tissues like brain (~1 kPa) [21,22]. The key advantage of ADA over pristine alginate is its capability to be functionalized with ligands, preferably proteins, to enhance cytocompatibility and cell attachment [23]. Free aldehyde groups of ADA can form Schiff’s bases with available amino groups of proteins. As a result, gelatin can be retained in the hydrogel matrix, which is otherwise prone to fast release and degradation [20]. In addition, a more homogeneous cell growth has been observed in ADA-GEL hydrogels in comparison to alginate-GEL hydrogels, emphasizing the potential of this matrix as a 3D cell culture microenvironment [24]. The protein binding properties of ADA can be utilized to modify the hydrogel with matrix proteins such as laminin [25]. However, such a modification has only been used in combination with oxidized hyaluronic acid and pre-adipocyte/adipocyte delivery application [25,26], not investigating its potential as a 3D matrix for neural applications. While ADA-GEL has shown promising properties for e.g., vascular and bone tissue TE [27,28,29], the application of ADA-GEL modified with laminin might ideally facilitate the development of neuronal networks due to its softness, degradability and incorporation of native cell adhesion motifs via crosslinking of gelatin and laminin.

**Figure 1:**
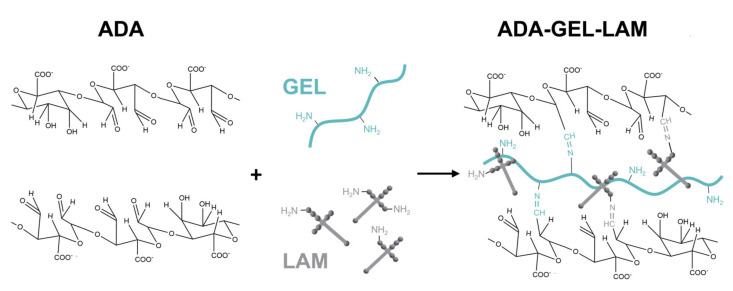
Schematic of ADA-GEL-LAM hydrogel. By oxidation of alginate, alginate di-aldehyde (ADA) is formed. The presence of aldehyde groups allows crosslinking with amine groups of proteins (gelatin, laminin) via nucleophilic attack of the amine on the aldehyde.

The aim of this study was to investigate laminin modified oxidized alginate-gelatin hydrogels (ADA-GEL-LAM) (Figure 1) as a 3D culture system to assess the influence of laminin addition on the differentiation and neuronal outgrowth of embedded hiPSC-derived neurospheres consisting of neural progenitor cells (hiNPC). The mechanical stiffness and microstructure of ADA-GEL-LAM hydrogels are characterized via nanoindentation and scanning electron microscopy techniques, respectively. Immunocytochemistry is used to analyze hiNPC differentiation into neurons in ADA-GEL and ADA-GEL-LAM hydrogels.

## 2. Materials and Methods

Sodium alginate (VIVAPHARM PH176 alginate, Ph. Eur.) was purchased from JRS PHARMA GmbH & Co. KG (Rosenberg, Germany). Ethylene glycol (≥99% purity) was bought from VWR Chemicals International (Radnor, PA, USA). PBS (#L1825/L1835) for cell culture was purchased from Biochrom (Berlin, Germany). Murine sarcoma basement membrane laminin (LAM) was purchased from Sigma (#L2020). All other chemicals were purchased from Sigma Aldrich if not otherwise noted.

### 2.1. Materials Synthesis

Oxidized alginate was synthesized by controlled oxidation of sodium using sodium metaperiodate (NaIO_4_, ACS reagent, ≥99.8%) as described earlier [20]. In brief, sodium alginate (10 g) was transferred to equal amounts (100 mL in total) of H_2_O:Ethanol (EtOH, absolute, EMSURE^®^ ACS regent, ≥99.9%) containing 9.375 mmol NaIO_4_. Oxidation of alginate was carried out for 6 h in the absence of light at room temperature (RT, 22 °C). The reaction was quenched using 10 mL ethylene glycol and stirred for additional 30 min. The dispersion was allowed to sediment for 5 min, the ethanol was decanted, and the ADA was transferred into dialysis tubes (MWCO: 6–8 kDa) and dialyzed for 7 days against ultrapure water (Type II, Merck MilliPore, Darmstadt, Germany). The final product was frozen for a minimum of 24 h and lyophilized using a freeze dryer (Alpha 1-2LD plus, Martin Christ, Osterode am Harz, Germany). The level of oxidation of the final ADA product amounted to ~19%.

### 2.2. Preparation of Hydrogels

For the preparation of the ADA stock solution, ADA was weighted inside a beaker with a stirring bar. PBS (w/o Ca^2+^/Mg^2+^) was added to create a 5% or 10% (*w*/*v*) ADA stock solution. The solutions were stirred at 200 rpm (SU1200 magnetic stirring plate, Sunlab^®^ Instruments, Mannheim, Germany) using a magnetic stirring bar at RT (5%) or 40 °C (10%) for ~3–4 h and the pH of the solutions (pH ~6.51) was adjusted to pH 7.4 using 0.1 M NaOH. The ADA solutions were subsequently filtered with pre-warmed 0.45 µm PVDF filters (37 °C) to allow in vitro cell culture. The ADA was stored at 4 °C until further use.

Gelatin was weighed inside a beaker with a stirring bar already inserted and supplemented with an appropriate volume of PBS (w/o Ca^2+^/Mg^2+^) to create a 5% or 20% (*w*/*v*) gelatin stock solution. The gelatin was dissolved by stirring at 200 rpm (SU1200 magnetic stirring plate, Sunlab^®^ Instruments, Mannheim, Germany) using a magnetic stirring bar at 40 °C for ~3–4 h and the pH of the solutions (pH ~6.51) was adjusted to pH 7.4 using 0.1 M NaOH. The gelatin solutions were heated to 50 °C prior to the filtration with pre-warmed 0.45 µm PVDF filters (37 °C) to allow for suitability for in vitro cell culture. The gelatin was stored at 4 °C until further use.

ADA-GEL was prepared by mixing ADA and GEL solutions for 10 min at 37 °C in a volume ratio of ADA:GEL 50:50 to gain ADA-GEL hydrogel precursor solution with a final concentration of 2.5% (*w*/*v*)/2.5% (*w*/*v*) ADA-GEL.

ADA-GEL-LAM was prepared by adding 100 µL LAM stock (c_stock_ = 0.1%) to 250 µL GEL (10% *w*/*v*) and 150 µL PBS, followed by mixing with 500 µL ADA (5% (*w*/*v*)) for 10 min at 37 °C. The final hydrogel concentrations were 2.5% (*w*/*v*)/2.5% (*w*/*v*)/0.01% ADA-GEL-LAM.

ADA-LAM was prepared similarly by adding 100 µL LAM stock to 400 µL PBS and 500 µL ADA (5% *w*/*v*), with final concentrations of 2.5% (*w*/*v*) ADA/0.01% LAM.

Hydrogel specimen were fabricated by casting hydrogel precursor solutions (200 µL) into custom-made cylindrical PDMS molds (diameter d = 10 mm), allowing the solutions to set at RT for 10 min. Following this, the hydrogels were crosslinked using 0.1 M CaCl_2_ solution for 10 min. Hydrogels were removed from the molds and crosslinked for another 15 min in 0.1 M CaCl_2_ to ensure homogenous crosslinking throughout the hydrogels prior to the material’s characterization.

### 2.3. Hydrogel Characterization

To investigate the influence of laminin on hydrogel stiffness, nanoindentation of ADA-GEL and ADA-GEL-LAM hydrogels was performed. The gels (*n* ≥ 3) were immersed in Hanks buffered salt solution (HBSS) to avoid dehydration during the measurement and indented directly after immersion as technical triplicates (three indentations each sample) with a cantilever (tip radius: 24.5 µm; 4.6 N/m; Optics II, HV Amsterdam

Amsterdam, NL using a nanoindentation setup (PIUMA Nanoindenter, Optics II, HV Amsterdam, The Netherlands). Indentation loading curves were recorded and the effective Young’s Modulus was calculated using the Oliver & Pharr method [30] implemented in the PIUMA software. The effective Young’s Modulus is reported as it accounts for the elastic displacements which occur in the specimen (Young’s modulus E, Poisson’s ratio ν) and the indenter tip (E_indenter_, ν_indenter_) [31], while the Poisson’s ratio of the hydrogel is unknown. All hydrogel samples were assessed at 22 °C (RT) and 37 °C.

The chemical composition of ADA, ADA-GEL, ADA-GEL-LAM, and ADA-LAM was assessed using attenuated total reflectance-Fourier transformed infrared spectroscopy (ATR-FTIR). Hydrogel samples were frozen and freeze-dried using a liophilizer (LD12 plus, Martin Christ, Osterode am Harz, Germany). Dry samples were then assessed and attenuated total reflectance spectra were recorded using an infrared spectrometer (Nicolet 6700, Thermo Scientific, Waltham, MA, USA).

The hydrogel’s microstructure was assessed using scanning electron microscopy (SEM). Samples were washed with PBS and fixed using 3% glutaraldehyde (#G5882) in PBS, 1 h at RT, and later stored at 4 °C until drying. Dehydration was employed by an ascending ethanol series followed by critical point drying in liquid CO_2_. Prior to SEM imaging using the FEI-Philips XL30 ESEM FEG, a 12.5 nm palladium/gold (Science Services, Munich, Germany) film was deposited on the insulating surfaces using a Leica EM SCD500 high vacuum sputter coater to avoid sample charging.

### 2.4. Cell Culture and Maintenance

Human induced pluripotent stem cell (hiPSC) culture and neural induction were adapted from a previously published study [32]. In brief, hiPSCs were purchased (iPS(IMR90)-4; WiCell, Madison, WI, USA) and cultivated on Matrigel coated dishes (hESC-qualified matrix, LDEV-free, #354277, Corning, Corning, NY, USA) in mTeSR1 medium (#05850, StemCell Technologies, Cologne, Germany). After pre-incubation with 1 µM ROCK-inhibitor (Y-27632, #1254, Tocris Biosciences, Bristol, UK) in mTeSR1 for 1 h, fragmented colonies were transferred onto a poly(2-hydroxyethly methacrylate) (#P3932, Merck, Darmstadt, Germany)-coated dish and to neural induction medium (see Supplementary Table S1) supplemented with 1 µM ROCK-inhibitor. The medium was changed three times per week. Upon first medium change, ROCK inhibitor was excluded from the medium. Seven days after the start of the induction, 10 ng/mL recombinant human FGF (#233-FB, R&D Systems, Minneapolis, MN, USA) was added to the culture medium. Upon day 21, formed neurospheres (hiNPC, hiPSC-derived neural progenitor cells) were cultivated in proliferation medium (Supplementary Table S1). HiNPCs were controlled by FACS as already described [32,33]. Spheres were chopped approximately every week to 0.2 µm using a McIlwain Tissue Chopper (Ted Pella, Redding, CA, USA). Cell viability was measured by the ability of cells to reduce resazurin to fluorescent resorufin using the CellTiter-Blue (#G8080, Promega, Madison, WI, USA). The cytotoxicity was verified by measuring the extracellular lactate dehydrogenase (LDH), which is based on the LDH release from dead and apoptotic cells and is indicative for cytotoxicity, using the CytoTox-ONE Homogeneous Membrane Integrity assay (#G7890, both Promega, Madison, WI, USA). All assays were performed in accordance with the manufacturer’s instructions. The amount of cells in 2D, indicated in the instructions, was seeded onto laminin-coated surfaces, as well as permeabilized 2D samples, which were prepared using Triton-X to serve as positive and negative controls. A LIVE/DEAD Viability/Cytotoxicity Kit for mammalian cells was used to visualize living and dead cells (#L3224, Invitrogen, ThermoFisher Scientific, Waltham, MA, USA) simultaneously using 0.6 µM Calcein-AM and 0.1 µM EthD-1. Samples were incubated in the dark for approximately 45 min at 37 °C and 5% CO_2_ and subsequently analyzed using the Cellomics Array Scan (ThermoFisher, Waltham, MA, USA).

### 2.5. Preparation of Sphere-Laden Hydrogels Subsection

Pre-filtered ADA and GEL solutions were heated to 37 °C prior to hydrogel preparation. GEL (20% (*v*/*w*)) and laminin (0.1% (*w*/*v*)) stock solutions were mixed in equal parts to create a GEL-LAM precursor gel (10% gelatin, 0.04% laminin). The pre-warmed ADA (10% (*w*/*v*)) was added in a 1:1 (*v*/*v*) ratio to create an ADA-GEL-LAM precursor gel solution (5% ADA, 5% gelatin and 0.02% laminin). The hydrogel solution was stirred for 10 min at 200 rpm (SU1200 magnetic stirring plate, Sunlab^®^ Instruments, Mannheim, Germany) and 37 °C using a magnetic stirring bar. The previously prepared cell suspension of 2 × 10^4^ spheres/mL (one 0.1 mm sphere equaled approximately 1 × 10^3^ cells) was carefully mixed in equal parts with the ADA-GEL-LAM precursor gel, creating a cell-laden hydrogel solution with final concentrations of 2.5% ADA, 2.5% GEL, 0.01% laminin, and 1 × 10^7^ cells/mL. The ADA-GEL and ADA-LAM hydrogels were prepared in a similar manner by generating ADA-GEL (5% ADA, 5% GEL) and ADA-LAM (5% ADA, 0.02% laminin) precursor gels from the 5% (*w*/*v*) ADA and 5% (*w*/*v*) GEL stock solutions. The hydrogels were allowed to mix for 10 min at 37 °C (200 rpm, SU1200 magnetic stirring plate, Sunlab^®^ Instruments, Mannheim, Germany) prior to mixing equal volumes of cell suspension (2 × 10^7^ cells/mL) and hydrogel (1:1 *v*/*v*) with the hydrogels. The final hydrogels consisted of ADA-GEL (2.5% ADA, 2.5% GEL) and ADA-LAM (2.5% ADA, 0.01% laminin) with a cell density of 1 × 10^7^ cells/mL, respectively. Cell-laden hydrogels were crosslinked for 10 min at 37 °C in CaCl_2_ solution (90 mM).

### 2.6. Cytocompatibility Study

To investigate the cytocompatibility of ADA-GEL, ADA-GEL-LAM, and ADA-LAM hydrogels for the culture of hiNSC derived neurospheres, the embedded cells were cultured for 7 days inside the hydrogels in differentiation medium (Supplementary Table S1). Cell viability was assessed using a resazurin conversion CellTiter-Blue (#G8080) assay. Cytotoxicity was investigated by LDH release using a CytoTox-ONE Homogeneous Membrane Integrity assay (#G7890, both Promega, Madison, WI, USA) according to the manufacturer’s instructions. LIVE/DEAD Viability stainings were performed using 0.6 µM Calcein-AM and 0.1 µM EthD-1 (#L3224, Invitrogen). Samples were incubated for 45 min at 37 °C and 5% CO_2_ and analyzed using the Cellomics Array Scan (ThermoFisher). Cells cultured in 2D on LAM-coated substrates as well as ADA-GEL served as positive 2D and 3D controls to LAM-supplemented ADA-GELs.

### 2.7. Immunocytochemistry

Samples were washed with pre-warmed DPBS and fixed with 4% paraformaldehyde (#P6148, Sigma-Aldrich, St. Louis, MO, USA) in DPBS (#14040, ThermoFisher Scientific, Waltham, MA, USA) for 1 h at RT. After fixation, samples were washed three times with DPBS for 10 min. Hydrogel samples were incubated in 10% (*v*/*v*) goat serum (#G9023)/0.1% (*v*/*v*) Triton X-100 (#T8787)/PBS, and TUBB3 antibody (1:200, rabbit polyclonal, #T2200, all Sigma-Aldrich, St. Louis, MO, USA), at 4 °C overnight. Specimens were washed three times for 2 h each with DPBS and subsequently incubated with anti-rabbit-Alexa546 (1:500, #A11010, Invitrogen), Phalloidin-Alexa488 (1:70, #A12379, ThermoFisher), and Hoechst (1%, bis-benzimide H33258, #B1155, Sigma) in 2% goat serum/PBS over night at 4 °C. Subsequently, samples were washed three times for 2 h each in PBS at RT and directly imaged by a confocal laser scanning microscope (CLSM, TCS SP8, Leica Microsystems, Wetzlar, Germany) using the objectives HC PL FLUOTAR 5x/0.15 DRY, HC PL APO CS2 10x/0.40 DRY, and HC PL APO CS2 20x/0.75 DRY. Maximum intensity projections of recorded z-stacks were constructed using Fiji Image J 1.52p [34]. ADA-GEL hydrogel served as control to LAM-supplemented ADA-GEL hydrogels.

### 2.8. Statistical Analysis

Data were analyzed using Graphpad Prism^TM^ (GraphPad Software Inc., San Diego, CA, USA) and Origin^®^Lab (OriginLab Corporation, Northampton, MA, USA) software. Means ± standard deviations (SD) of individual experiments are displayed (*n* ≥ 3 (nanoindentation), *n* = 3 (cell culture studies)). Statistical analysis was performed by one-way ANOVA (Origin 2019, OriginLab Software, v9.6.0.172), using a post-hoc Bonferroni’s pairwise mean comparison between multiple groups. Image analysis was performed using ImageJ (ImageJ Software, https://imagej.nih.gov/ij/, accessed on 20 October 2019).

## 3. Results

### 3.1. Material Characterization

**Figure 2:**
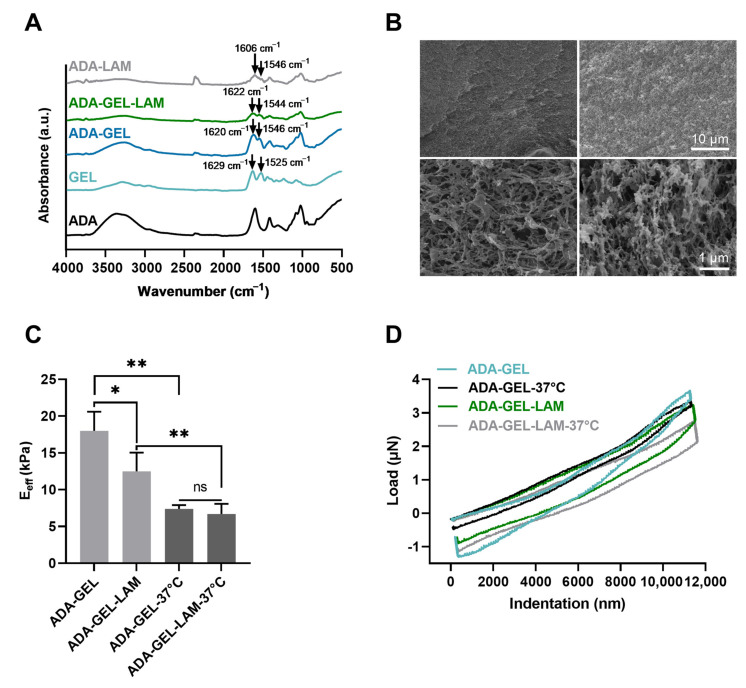
Material characterization. (**A**) Fourier transformed infrared spectroscopy (FTIR) analysis, absorbance spectra of ADA, GEL, ADA-GEL, ADA-GEL-LAM, and ADA-GEL hydrogels. (**B**) Scanning electron microscopy images of ADA-GEL and ADA-GEL-LAM hydrogels. Both hydrogels feature micro porosity (bottom row). (**C**) Nanoindentation of ADA-GEL and ADA-GEL-LAM showing the effective Young’s modulus (E_eff_) of the hydrogels at 22 °C and 37 °C. Data are displayed as mean ± SD. (**D**) Qualitative load-indentation behavior of the hydrogels. Statistically significant differences of means analyzed using one-way ANOVA with * *p* < 0.05, ** *p* < 0.01, not significant (ns, *p* ≥ 0.05). Scale bar 1–10 µm.

By the addition of gelatin and laminin to ADA, FTIR analysis showed the presence of amine I (1620 cm^−1^) and amine II (1546 cm^−1^) peaks in ADA-GEL, ADA-GEL-LAM, and ADA-LAM spectra, characteristic for the presence of primary and secondary amine groups of gelatin and laminin protein [20,35] inside the hydrogels (Figure 2A). The peak shift of primary amines I and II (GEL spectrum) from 1629 cm^−1^ and 1525 cm^−1^ towards lower (1620 cm^−1^) and higher (1546 cm^−1^) wave numbers in ADA-GEL and ADA-GEL-LAM indicate Schiff’s base formation of gelatin and laminin with ADA [20]. Peaks at ~1546 cm^−1^ in ADA-LAM are indicative of the presence of laminin in comparison to the pristine ADA spectrum [35]. Both, ADA-GEL and ADA-GEL-LAM formed hydrogels featuring a microporous microstructure, which was confirmed via SEM analysis (Figure 2B). ADA-GEL and ADA-GEL-LAM present a similar microstructure and porosity (Figure 2B).

Indentation analysis showed that by the addition of laminin to ADA-GEL, the hydrogel stiffness decreased from approximately 17 ± 2 kPa to 12 ± 2 kPa (Figure 2C). Heating of the samples to 37 °C to mimic cell culture conditions revealed a stiffness reduction of both ADA-GEL and ADA-GEL-LAM hydrogels, by almost 60% (ADA-GEL) and 35% (ADA-GEL-LAM) (Figure 2C), resulting in stiffnesses in the range of ~5–7 kPa. The qualitative nanoindentation force-penetration behavior was similar for all hydrogels and independent of laminin addition (Figure 2D), with ADA-GEL-LAM at 37 °C leading to slightly less stiff hydrogels in comparison to ADA-GEL. Statistical analysis indicated no significant differences in mean stiffness of ADA-GEL and ADA-GEL-LAM.

### 3.2. Cytocompatibility of ADA-GEL-LAM Hydrogels

**Figure 3:**
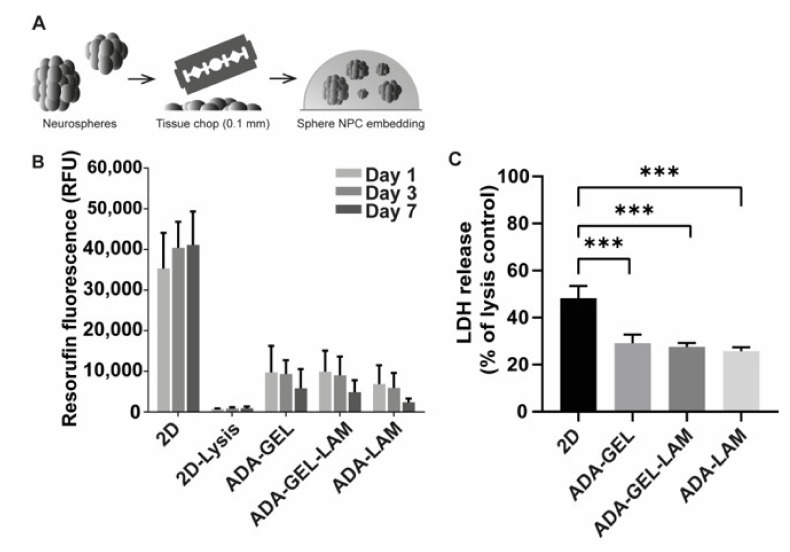
Cell viability and toxicity of iPSC-derived NPCs. (**A**) Cell embedding scheme. Neurospheres were freshly chopped to 0.1 mm, mixed with ADA-GEL-X precursor at a density of 10,000 spheres/mL (corresponds to 1 × 10^7^ cells/mL), crosslinked, and maintained in differentiation medium. (**B**) Viability of neurospheres in the hydrogels measured via resazurin reduction to resorufin. Data shown as relative fluorescence units (RFU). 2D as positive and negative (2D lysis, TritonX-100 treated) controls. (**C**) Cytotoxicity assessment via LDH release after one day of incubation in differentiation medium, normalized to 2D TritonX-100 treated lysis control (*n* = 3 with 4 technical replicates; *** *p* < 0.001 compared to 2D control).

Neurospheres were generated from hiPSC by neural induction as described earlier [32] and subsequently embedded into ADA-GEL, ADA-GEL-LAM, and ADA-LAM hydrogels after chopping neurospheres to 0.1 mm and mixing with the hydrogel precursor (Figure 3A). Cross-linked cell-laden hydrogels were further incubated in cell-differentiation medium to start neuronal differentiation. In order to assess the compatibility of the hydrogels with the neurospheres, cell viability was measured by resazurin reduction to fluorescent resorufin on days 1, 3, and 7 (Figure 3B).

**Figure 4:**
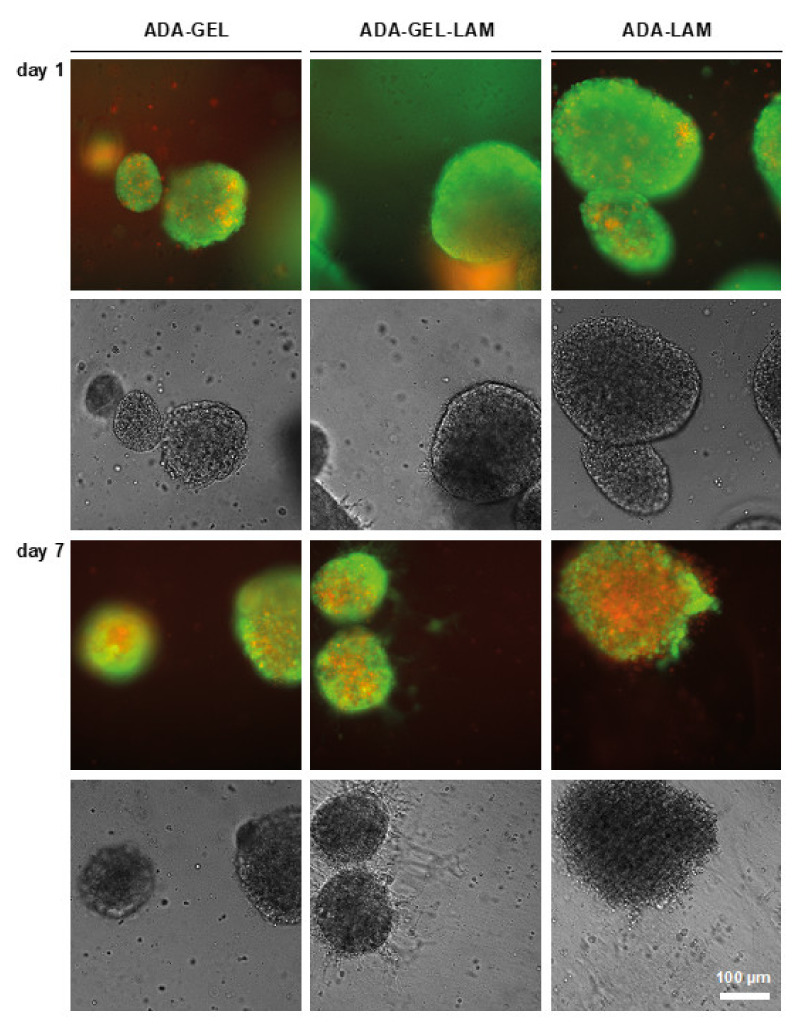
LIVE/DEAD staining of neurospheres in ADA-X hydrogels. Neurospheres were embedded in the indicated hydrogels and cultivated in differentiation medium. Samples were stained with Calcein-AM (LIVE, green) and Ethidium-homodimer (DEAD, red) and analyzed by light-microscopy in fluorescence and brightfield mode. Cell outgrowth was indicated in ADA-GEL-LAM (white arrows). Scale bar 100 µm.

Fluorescence intensities in all tested ADA-hydrogels was approximately 30% of the 2D control at day one. From the day 1 samples, LDH release as a readout for cytotoxicity was measured (Figure 3C). Cytotoxicity was above 40% of 2D lysis controls in 2D samples, indicating that the cell preparation induces high cell death. In hydrogel samples LDH release was approximately 30% of the 2D lysis controls. Cell viability slightly decreased over the time period of seven days for all tested hydrogels. However, the detected fluorescence was lower for ADA-LAM compared to the other materials (Figure 3B) despite the similar LDH release measured for all conditions (Figure 3C). To visualize the neurospheres in the hydrogels and to investigate the cell viability in situ, LIVE/DEAD fluorescence microscopy analysis was used (Figure 4). One day after hydrogel preparation, few dead (red stained) cells were visible outside of the embedded neurospheres, irrespective of the hydrogel composition. In ADA-GEL-LAM, protrusions from spheres appeared, which were visible in brightfield phase contrast images. At day seven, the observed protrusions or outgrowths of cells had increased. In addition, LIVE staining indicated viable cells in the observed protrusions, indicating cells actively migrating out of the spheres after seven days.

In contrast, for ADA-LAM without the presence of gelatin, the core of the neurospheres exhibited a high degree of necrosis, with spheres seeming to disintegrate as indicated by the red EthD-1-stained cells. In phase contrast images, the defined interface between the neurospheres and the bulk hydrogel vanished compared to ADA-GEL or ADA-GEL-LAM hydrogels.

### 3.3. Neurospheres Differentiate into Neurons

**Figure 5:**
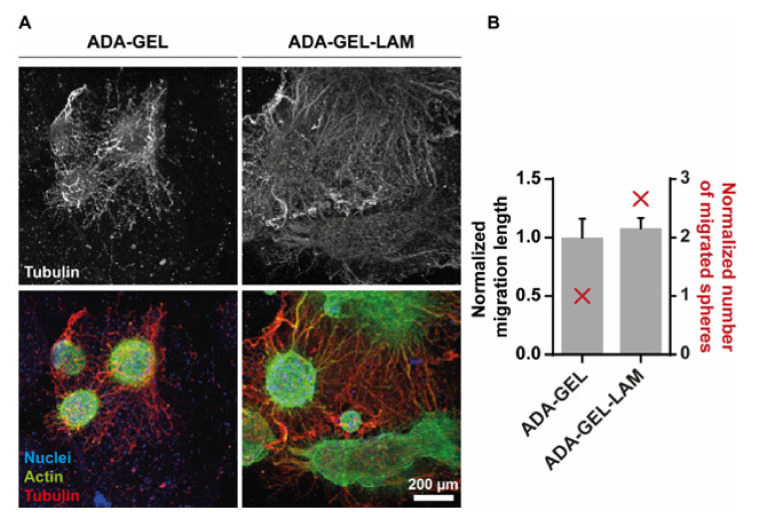
Laminin supports neuronal migration out of neurospheres. (**A**) Confocal fluorescence microscopy of immunostainings for tubulin-β-III (red, Alexa546), filamentous actin (green, Phalloidin-Alexa488) and nuclei (blue, Hoechst) after 14 days of gel differentiation. The upper panel displays the tubulin-β-III positive neuronal outgrowth only. Representative maximum intensity projections are shown in the lower panel. (**B**) Quantification of the migration distance (black), measured from the edge of the sphere core to the distal end, normalized to the length of ADA-GEL. Number of spheres showing migration, normalized to ADA-GEL (red). Scale bar 200 µm.

Cell differentiation was further monitored after a time period of 14 days using confocal laser scanning fluorescence microscopy. Filamentous (F-)actin as well as the neuronal marker tubulin-β-III (TUBB3) were labeled by phalloidin and specific antibodies, respectively (Figure 5). ADA-LAM showed dispersed spheres with cells appearing necrotic as indicated by cell viability LIVE/DEAD stainings, similar to earlier time points (data not shown). However, neurospheres in ADA-GEL were interconnected via neuronal paths, indicated by TUBB3-positive neurites (Figure 5A, red). Moreover, in laminin-crosslinked ADA-GEL-LAM, the cellular outgrowth of spheres was stronger and omnidirectional (Supplementary Videos S1 and S2), suggesting laminin-supported cell migration (Figure 5A). This observation was quantified by determination of the number of spheres with migrated cells and measurement of the migration distance.

Although migration distance showed no significant difference, laminin presence stimulated cell migration out of the spheres by 2.7-fold (Figure 5B). The results thus suggest that laminin supports cell differentiation and migration.

## 4. Discussion

Our work demonstrates for the first time that ADA-GEL hydrogel is cytocompatible with hiPSC-derived neurospheres, opening the possibility of the application of this gel base for 3D tissue modeling. Both neuronal outgrowth and migration in 3D are supported by ADA-GEL hydrogels and are further enhanced in the presence of laminin, resulting in dense neuronal differentiation in 3D. Although different alginate-based hydrogels were previously used for neural applications—including rodent spinal cord, neurite outgrowth activity and cell-matrix-adhesion [36,37,38,39]—the application of oxidized alginate-gelatin-laminin hydrogels for central nervous system neuronal differentiation from hiPSC-derived NPC represents a novel approach in the field.

We suggest the following functions of each material inside ADA-GEL-LAM: ADA provides the main 3D hydrogel environment by ionic crosslinking using Ca^2+^ ions. GEL may offer initial cell adhesion motifs for neurosphere attachment, as shown for other cell types in ADA-GEL [20,32,40,41]. The availability of amine groups by GEL facilitates Schiff’s base formation with aldehyde groups of ADA, saturating the aldehyde groups in ADA, which is indicated by the higher cell viability inside ADA-GEL-LAM in comparison to ADA-LAM (Figure 3 and Figure 4). ADA and GEL degrade over time [20], providing space for neuron outgrowth. LAM provides integrin binding motifs for hiNPC similar to human neural stem cell (hNSC)-derived neurons [42,43] and PC-12 cell neuron outgrowth [38], thus providing adhesion and guidance for neuron outgrowth in comparison to pristine ADA-GEL (Figure 5). In addition, amine groups of LAM crosslink to aldehyde groups of ADA via Schiff’s base formation [25]. The network formation of LAM at 37 °C contributes to the ADA-GEL-LAM hydrogel network supplying an environment for cell adhesion and migration. LAM seems to be the key contributor to the enhanced 3D differentiation of hiNPC’s inside the ADA-GEL-LAM hydrogels.

The ECM is capable of influencing cellular differentiation by activating different downstream signaling pathways through interaction with cellular adhesion receptors [44]. Laminin influences cellular attachment and differentiation in multiple native tissue environments [45,46]. It is expressed during the development of the ventricular zone in rodents, which represents the neural stem/precursor cell niche in the developing cortex [43,47,48]. In the early human developing brain, laminin is expressed in the cortex after approximately 17 weeks of gestation [43,49] and was found to be involved in the neuronal differentiation of cultured neural tube neurons [50]. As a result, laminin may represent a key ECM component influencing neural differentiation among other cytokines and stimuli. Laminin also impacts the differentiation of hNSC in vitro [42,51,52] involving the interplay of laminin with different growth factors [52]. An increased number of neurons differentiated from hNSC on laminin-coated substrates, which has been associated to laminin-binding integrins α3, α6, α7, β1, β4 found on the surface of hNSCs [43]. Our results on laminin addition to ADA-GEL hydrogels, which increased 3D neuron outgrowth of hiNPC-derived neurospheres, are in accordance with those findings. Laminin peptides, and here especially the IKVAV sequence, also impact neural cell differentiation in vitro [44,51,53], providing a more cost-efficient solution than using the whole laminin protein. IKVAV-functionalized nanofibers were shown to amplify the presence of bioactive IKVAV epitopes in comparison to 2D control substrates [51], while IKVAV increased migration, attachment, and differentiation of hNSC into neurons in polyethyleneglycol (PEG) hydrogels [44] and enhanced neural migration of primary hNPC in functionalized hydrogels in 3D [53]. In sum, evidence from the literature highlights the interplay of neuronal stem cells by laminin interaction, capable of influencing neuronal differentiation efficiency, neuronal cell adhesion, and migration. We add to this knowledge base by demonstrating the positive impact of laminin for neuronal differentiation and outgrowth in 3D ADA-GEL-LAM hydrogels.

Peak shifts of amine I and amine II peaks in FTIR analysis from 1525 cm^−1^ to ~1546 cm^−1^ and 1629 cm^−1^ to 1620 cm^−1^ indicate the formation of a Schiff’s base (imine bond) [20] in ADA-GEL-LAM and ADA-LAM hydrogels, inducing the crosslinking of laminin and GEL protein components to the ADA polysaccharide. Mechanical assessments showed that the stiffness of ADA-GEL decreased with laminin addition, with a relatively higher decrease in stiffness of ADA-GEL in comparison to ADA-GEL-LAM once incubated at 37 °C (Figure 2). This behavior might be associated to laminin crosslinking at 37 °C, which counteracts the stiffness loss due from the release of excess gelatin and ADA-GEL degradation after incubation [20,27,54]. Previous studies have shown a decrease in hydrogel stiffness with increasing laminin concentration for laminin-conjugated PEG hydrogels [55]. The addition of laminin may lower the initial stiffness of ADA-GEL by sterically hindering the formation of a dense ADA-GEL network. However, the reduction of stiffness can be in favor for neuron growth, due to its higher similarity to brain tissue stiffness (<5 kPa) [21]. An increased neuronal stem cell proliferation and enhancement of neuronal βIII-tubulin markers has been shown in soft hydrogels (~100 Pa–1 kPa) compared to stiffer hydrogels (10–20 kPa) [56]. Due to ADA-GEL degradation [20], a further decrease in hydrogel stiffness after day 14 can be expected for the ADA-GEL-LAM hydrogels investigated here, which may result in a lower stiffness than measured initially at 37 °C (Figure 2). A decrease in stiffness during incubation may explain the observed differentiation despite initial ADA-GEL-LAM hydrogel stiffness values of >1 kPa. ADA-GEL hydrogels degrade over time by a controlled release of GEL [20]. Therefore, we assume that this process also takes place in ADA-GEL-LAM hydrogels reducing their stiffness during incubation. In addition, hydrogel degradation was shown to be a crucial factor for the maintenance of neuronal progenitor stemness in 3D [57]. As a result, the ADA-GEL-LAM hydrogel stiffness is likely to adapt closer to brain tissue stiffness over incubation time, possibly enabling the observed neuronal migration by hydrogel degradation. The stiffness of 2.5% ADA—2.5% GEL hydrogels at 37 °C were reported to be in the range of 5 kPa [54], which is in accordance with the 2.5% ADA—2.5% GEL—0.01% LAM hydrogels here. Enzymatically crosslinked GEL-LAM hydrogels with a bulk stiffness of ~8.4 kPa (surface elastic modulus: ~24.1 ± 12.9 kPa, via AFM) facilitated the growth of hiPSC derived spinal spheroids [58]. While the cells detached on enzymatically crosslinked GEL substrates after two days [58], the combination of GEL and LAM lead to sustained cell attachment and growth [58]. We confirm the increased growth of hiNPC-derived neurospheres in GEL-LAM supplemented ADA in comparison to pure ADA-LAM, which is in accordance with that study. In sum, the ADA-GEL-LAM hydrogels presented here are in an initial stiffness range shown suitable for neuronal cell culture earlier. As the uncertainty of the mechanical properties and degradation of the ADA-GEL-LAM hydrogels over incubation time are one limitation of this study, we will assess the degradation of ADA-GEL-LAM and corresponding effects on the mechanical properties and hiNPC response to the hydrogels in future investigations.

ADA-LAM showed higher cytotoxicity in LIVE/DEAD staining analysis as well as disintegration of neurospheres in phase contrast images compared to ADA-GEL-LAM (Figure 3 and Figure 4). The low laminin concentration (0.01%) might not have yielded complete saturation of aldehyde groups of ADA by Schiff’s base crosslinking. Consequently, un-crosslinked aldehyde groups could act as a cytotoxic fixative similar to e.g., formaldehyde [59]. In addition, LAM-peptide AG73 interacts with cell syndecan transmembrane-proteins by ionic interaction of the LAM-peptide with the syndecan’s heparansulfate domain [38,60]. The anionic properties of ADA in the ADA-LAM system may counteract the LAM-heparansulfate interaction [38]. In contrast, the cationic properties of Typ A GEL at pH 7.4 (isoelectric point pH ~8–9) in ADA-GEL-LAM may lead to an enhanced LAM-heparansulfate interaction. This may further explain the increased cell survival in ADA-GEL-LAM besides the integrin binding sites offered by GEL [40]. Hence, the ADA-LAM system would require additional engineering to yield hydrogels of LAM concentration and oxidation degree tuned to ensure complete crosslinking of aldehyde groups with LAM. However, the overall lower resorufin adsorption measured in gels compared to 2D cultures without gels is likely not due to lower cell viability, but the result of assay compounds not diffusing into the gels easily. This notion is also supported by the lack of differences between ADA-GEL-LAM and ADA-LAM in this assay (Figure 3). Hence, the CellTiter-Blue assay is not well suited for the viability assessment of neurospheres embedded in hydrogels. Here, LIVE/DEAD stainings are preferred.

The use of hydrogels for neural cell applications, i.e., neuronal tissue regeneration, has been reviewed previously [61], identifying various LAM-modified hydrogels [55,62,63,64] among other modifications to be promising matrices for the promotion of neurite outgrowth, a process which is necessary for neuronal network formation. Exemplary, hyaluronic acid-laminin hydrogels facilitate in vivo migration of neural progenitor/stem cells (NPSC) [65]. Collagen-based hydrogels were modified using LAM or LAM-derived polypeptides, demonstrating that LAM promotes superior neural cell growth and neural differentiation in 3D in vitro [66,67,68]. Laminin- and poly-L-ornithine (PLO)-modified alginate improved axonal growth, neurite outgrowth, and cell adhesion of rat dorsal root ganglia cells (DRG), and increased regenerative capacity and cell-ingrowth in PLO/LAM hydrogel microchannels was observed [36]. Similarly, improved nerve outgrowth of DRG by the use of laminin-alginate hydrogel capillaries has been reported [37]. Alginate functionalized with three different laminin peptides (A99, AG73, EF1zz) supported neurite outgrowth of rat pheochromocytoma (PC12) cells dependent on alginate and laminin peptide concentrations [38], while not triggering cell adhesion of fibroblasts [39]. Due to the different cells, gels, applications, and types of laminin, it is challenging to derive an overall conclusion from these studies. Yet, the data indicate that laminin in general supports neural cell performance in 3D cultures. In contrast to the present study, previous studies utilized oxidized alginate conjugated specific mouse LAM-based cys-peptides, like A99a, EF1XmRk, and A99T [69], or chitosan [70], instead of GEL and LAM which were used here. The advantages of the ADA-GEL-LAM system presented here are based on its cost-efficiency and ease of production, as it is based on mainly ADA and GEL (2.5%/2.5%) with only a small amount of supplemented LAM (0.01%), which already supported hiPSC-derived dense neuronal differentiation and migration in 3D. This ADA-GEL composition was previously used for 3D bioprinting [19], yet without the addition of laminin. The applicability of ADA-GEL-LAM for the 3D culture of hiPSC-derived neurospheres and the suitability of a 3D printable ADA-GEL based hydrogel composition [19] for the culture of neurospheres provides an exciting ground for future neurosphere-based 3D TE. The results confirm that ADA-GEL-LAM represents a cost-efficient and promising in vitro hydrogel model system to study neuronal cells in 3D, with the potential to create 3D printed in vitro models in future research.

LAM-modified hydrogels seem to be promising for a variety of different biomedical applications besides neural cultures. Exemplary, functionalized alginate wound dressings using laminin-derived peptides promote wound healing [71], oxidized alginate-laminin hydrogels have a potential as autologous fat grafting models [25,26], and ADA-LAM has shown to be a suitable carrier matrix for pre-adipocytes [72]. A selection of laminin peptides was identified to enhance pancreatic islet function in alginate-based microcapsules [73] and LM-111-containing fibrin hydrogels enhanced VEGF production and reduction of IL-6 of C2C12 myoblasts relevant for skeletal muscle engineering [74]. In contrast to these studies [25,26,75] and other ADA-based ADA-LAM systems [36,38,69], we here use a distinct cell type, i.e., hiNPC-derived neurospheres, different laminin, and GEL as an additional hydrogel component. Hence, the ADA-GEL-LAM hydrogels developed in this study might provide a benefit for the development and optimization of various TE applications.

## 5. Conclusions

We demonstrate that adding laminin as an important matrix component to oxidized alginate-gelatin hydrogels can be an effective tool to enhance cell adhesion, migration, and differentiation of embedded iPSC derived neurospheres. ADA-GEL-LAM showed lower cytotoxicity than ADA-LAM, associated to the complete crosslinking of aldehyde groups of ADA with excess amine groups of GEL and to the higher concentration of RGD-sequence containing GEL proteins in the matrix. All hydrogels showed initial stiffness values of approximately 5 kPa close to the relevant range of native brain tissue. Initial neuron out-growth was observed after 7 days in vitro, with higher spatial distribution and neuron migration observed after 14 days for ADA-GEL-LAM in comparison to ADA-GEL. Our study suggests that ADA-GEL-LAM can be a promising and cost-effective 3D hydrogel system for neural cell culture, with the future option for 3D-bioprinting. Finding matrices for building neural cell models in 3D in vitro is crucial in light of TE. Such 3D-models with tissue-like properties are increasingly gaining attention in regenerative medicine, basic research, drug development, and toxicity testing [76]. They should be of human relevance, allow physiological processes, permit application-oriented processing like 3D bioprinting, and be cost-effective. The successful application of ADA-GEL-LAM hydrogels for 3D hiPSC-derived neural culture contributes to this fast developing field of TE by meeting the mentioned criteria to some extent. In future, the application of ADA-GEL-LAM hydrogels may thus aid in replacing and reducing animal experiments in accordance with the 3R principle [75].

## Data Availability

The data presented in this study are available on request from the corresponding authors.

## Supplementary Materials

The following are available online at https://www.mdpi.com/2227-9059/9/3/261/s1, Table S1: Cell culture media compositions utilized for neural induction and differentiation. Video S1: ADA-GEL-LAM (0.01% lam)—day 14. Video S2: 3D_System_Neurospheres_in_ADA-GEL-LAM.
