# Supplementary material for: Neuronal Differentiation from Induced Pluripotent Stem Cell-Derived Neurospheres by the Application of Oxidized Alginate-Gelatin-Laminin Hydrogels
Source: Biomedicines. 2021 Mar 5;9(3):261. doi: 10.3390/biomedicines9030261 (PMC8000907; doi:10.3390/biomedicines9030261)
Supplement: Supplementary file 1 [file biomedicines-09-00261-s001.zip › Table.pdf]

Supplementary Materials

Table 1. Cell culture media compositions utilized for neural induction and differentiation.

I. Neural induction of hiPSC	II. Neural differentiation	III. Proliferation
	Basic medium for all steps:<br>DMEM high glucose/Ham's F12 (2:1 ratio, #31966-021/#31765-027, Gibco),<br>1 x penicillin/streptomycin (PAN-Biotech),<br>ta	
1 x N2 Supplement (Gibco),<br>1 x B27 supplement (Gibco),<br>20% (v/v) Knockout serum replacement (#10828028, Gibco),<br>16 ng/mL EGF,<br>20 μM TGF-β RI kinase inhibitor VI (SB-431542, #616461),<br>0.5 μM BMP receptor antagonist (LDN-193189, #SML0559,<br>both Sigma-Aldrich, St. Louis, USA).<br><br>[+/- ROCK-inhibitor<br>+/- human FGF – see explanation in main text]	1 x N2 Supplement (Gibco),<br><br>for hiNPC only supplemented with:<br>1 x B27 supplement (Gibco),<br>100 U/mL Interferon-γ,<br>20 ng/mL neurotrophin-3<br>(both PeproTech, Germany),<br>300 μM dibutyryl-cAMP,<br>20 μM ascorbic acid,<br>5 mM creatine monohydrate (all Sigma Aldrich, St. Louis, USA)	1 x B27 supplement (Gibco),<br>20 ng/mL human recombinant epidermal growth factor EGF (#PHG0313, Gibco),<br><br>for hNPC/hiNPC only supplemented with:<br>human recombinant FGF (# 233-FB, 20 ng/mL, R&D Systems),
